# Proportion and factors associated with recent HIV infection in a cohort of patients seen for care in Italy over 1996-2014: Data from the ICONA Foundation Study cohort

**DOI:** 10.1371/journal.pone.0189045

**Published:** 2017-12-05

**Authors:** Silvia Nozza, Alessandro Cozzi-Lepri, Francesca Bai, Stefano Rusconi, Andrea Gori, Paola Cinque, Adriana Ammassari, Pietro Caramello, Giuseppe Tambussi, Antonella D’Arminio Monforte, Giulia Marchetti, on behalf of Icona Foundation Study Group

**Affiliations:** 1 Infectious Diseases Department, San Raffaele Scientific Institute, Milan, Italy; 2 Department of Infection and Population Health, Division of Population Health, UCL Medical School, Royal Free Campus, London, United Kingdom; 3 Department of Health Sciences, Clinic of Infectious Diseases, ASST Santi Paolo e Carlo, S. Paolo Hospital, University of Milan, Milan, Italy; 4 Clinic of Infectious Diseases, DIBIC Luigi Sacco, University of Milan, Milan, Italy; 5 Division of Infectious Diseases, ASST Monza-Brianza, San Gerardo Hospital, University Milano-Bicocca, Monza, Italy; 6 Clinic of Infectious Diseases, INMI Spallanzani Hospital, Rome, Italy; 7 Infectious and Tropical Diseases Unit, Amedeo di Savoia Hospital, Turin, Italy; University of New South Wales, AUSTRALIA

## Abstract

In Italy the prevalence of recent HIV infection (RHI) isn’t currently monitored. Early diagnosis is crucial to allow introduction of antiretroviral therapy (cART) in the recent phase of infection. We aimed to estimate the proportion and the determinants of RHI among patients enrolled in the ICONA cohort; we explored differences in the median time from HIV diagnosis to cART initiation and in the viro-immunological response between RHI and Less Recent HIV infections (NRHI). We included antiretroviral-naïve HIV-positive patients enrolled in the cohort with documented dates of HIV-negative and positive antibodies tests, grouped in RHI (estimated date of seroconversion within 12 months of enrolment) and NRHI. Proportion of RHI and the trend of this proportion by calendar period (1996–2014) were investigated (Chi-square test). Logistic regression analysis was employed to identify factors associated with RHI. The time from seroconversion to cART initiation was compared in RHI and NRHI overall and after stratification by calendar period (survival analysis). We finally explored the time from starting cART to HIV-RNA <50 copies/mL and to CD4+ gain ≥200 cells/mmc by Cox regression. HIV seroconversion could be estimated for 2608/12,616 patients: 981/2608 (37.6%) were RHI. Proportion of RHI increased in recent calendar periods and was associated with younger age, baseline higher HIV-RNA and CD4+ count. There wasn’t difference in the 2-year estimates of cART start between RHI and NRHI, regardless of calendar period. Rates and hazards of virological response were similar in RHI versus NRHI. RHI showed a 1.5-fold higher probability of CD4+ gain, also following adjustment for calendar period and cART regimen, and for age, HCV and smoking; the difference in probability was however attenuated after further controlling for baseline HIV-RNA and CD4+ T-cells. The increased proportion of RHI over time suggests that in recent years in Italy HIV infections are more likely to be detected earlier than before. The similar rates of cART introduction and viro-immunological response in RHI and NRHI probably reflect the efficacy of the modern cART regimens. An improvement of the prevention services is warranted to allow an early cART access, also in the perspective of therapy as prevention.

## Introduction

Recent HIV Infection (RHI) is defined by a negative HIV antibody test within 6/12 months of diagnosis [1–3]. Recently the CASCADE collaboration published the largest study of seroconverters cohorts from 25 countries to estimate the rates of immunological decline and survival in HIV-positive patients; they found that mean age at seroconversion was 31.1 years for 16373 patients and 6947 started cART. Lower CD4+ counts at seroconversion and higher mortality rates were reported in HIV-positive patients infected at an older age [4]. Early diagnosis is crucial to insure benefit for the individual due to early access to care and cART, especially now that immediate treatment is recommended for all patients, and to reduce HIV transmission at population level [5–7]. Two recent studies have demonstrated the health benefits of an early initiation of cART for asymptomatic HIV-infected patients with high CD4+ counts: when cART was immediately started instead of waiting until CD4+ count was <350 cells/mmc, there was a reduction of over 40% in the risk of death or AIDS defining disease [6, 8]. In particular, early treatment leads to better immune recovery [9, 10], HIV reservoir decline [11, 12] and reduction of new infections, considering the high rate of transmissions during RHI [2, 3, 13]. In Italy new HIV diagnoses are reported to the Healthcare System; public health surveillance captures new diagnoses irrespective of time of HIV infection. Given the lack of current monitoring of RHI prevalence in Italy, we aimed to use the ICONA Foundation Study cohort to estimate the proportion and determinants of HIV infections diagnosed during the recent phase over the period 1996 to 2014; RHI was defined as having an estimated date of seroconversion within 12 months from the date of enrolment in the cohort. We also explored the differences in the time from seroconversion to cART initiation and in viro-immunological response under treatment between RHI and less recent infections (non RHI, NRHI).

## Materials and methods

We conducted an observational retrospective longitudinal study over 1996–2014. We included untreated HIV-positive patients with documented dates of HIV-negative and positive antibodies tests enrolled in the ICONA Foundation Study cohort. The Icona Foundation Study cohort is an observational multicentre cohort that enrolls HIV-infected individuals who are antiretroviral-naïve at the time of enrolment. A detailed description of the cohort is reported elsewhere [14]. Patients are voluntary enrolled by physicians at the different centres in Italy participating in ICONA Study after signing an informed consent. This cohort was set up in January 1997 and currently includes data on patients enrolled at 51 infectious disease units in Italy.

Participants’ date of HIV seroconversion was estimated as the midpoint between the last available HIV-negative and the first available HIV-positive test. People with such a date recorded within 1 year (since the last negative HIV serological test) were defined as RHI, according to current definition [2]. The proportion of RHI was calculated for the following calendar periods: 1996–2000, 2001–2006, 2007–2009, 2010–2014. Because the rate of enrolment in the cohort has been varying over time, the group period classification was based on the quantile of the distribution of enrolments in the cohort in order to have similar denominators in each calendar period.

AIDS defining conditions were diagnosed according to CDC revised classification system in 1993 [15]. Patients with hepatitis co-infection were defined as HCV Ab or HBsAg positive subjects. The proportion of RHI at entry in the cohort and the trend of this proportion by calendar period of enrolment were investigated using a Chi-square test. Univariable and multivariable logistic regression analysis was employed to identify factors associated with RHI. All the variables associated with the outcome with a p value <0.01 in the univariable analysis were selected for inclusion in the multivariable model; we thus included the following factors in the multivariable analysis: calendar period of seroconversion, gender, age, mode of HIV transmission, AIDS diagnosis, CD4+ T-cells and HIV-RNA (measured at enrolment), HCV co-infection, site geographical position, employment status, smoking and blood glucose. Survival analysis techniques were used to compare the time from seroconversion to cART initiation in RHI and NRHI, overall and after stratification by calendar period of enrolment (Kaplan-Meier method with log-rank test). We investigated the proportion of patients achieving a HIV-RNA ≤500 copies/mL at 6 months (time window +3; +9 months) from the date of starting cART, stratified by RHI status and calendar period (missing values of HIV-RNA were excluded from the analysis). We have chosen the threshold of 500 copies/mL, instead of 50 copies/mL, because this was the lower limit of detection of the assay used at the sites in the early calendar periods. We finally explored the determinants of probability of virological success (time from cART start to confirmed HIV-RNA <50 copies/mL) and of CD4+ count recovery (time from cART start to CD4+ gain ≥200 cells/mmc) by univariable and multivariable Cox regression models. We also performed a sensitivity analysis to explore the probability of immunological recovery according to another definition: CD4+ gain of 200 cells/mmc or reaching a single CD4+ count >350 cells/mmc, which ever came first.

The study was approved by the Ethical Committee of all the Centers participating to the ICONA Foundation Study (see acknowledgments and Ethics Statement for the full name of the ethics committees that approved the study). All patients signed written informed consent. Statistical analyses were performed using SAS software package (SAS Institute).

## Results

### Proportion of Recent HIV Infections in the ICONA Foundation Study cohort

**Fig 1:**
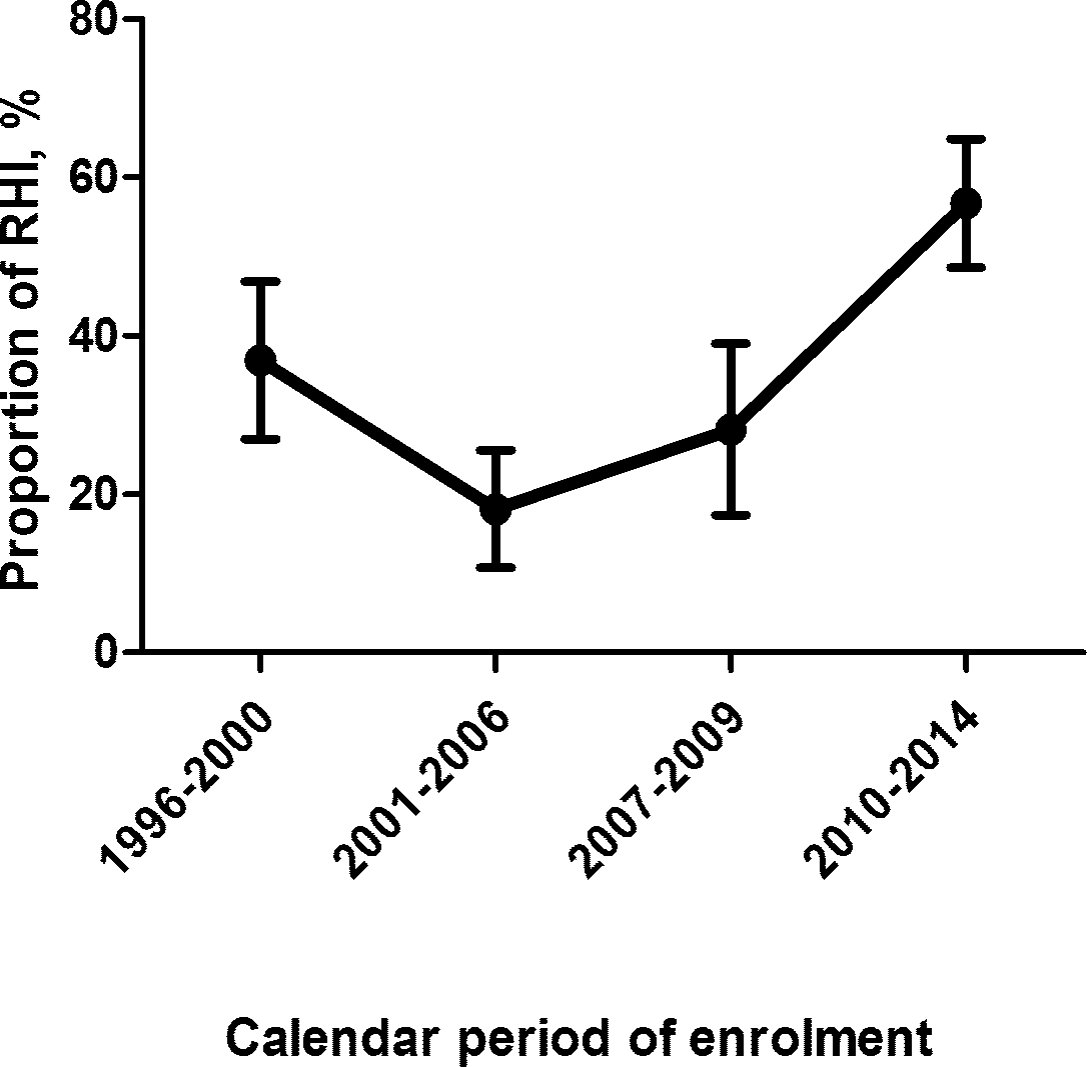
Proportion of Recent HIV Infections by calendar period of enrolment. The graph illustrates the proportion of Recent HIV Infections (RHI), defined as a positive HIV serological test within 12 months since the last negative one, according to calendar period of seroconversion (1996–2000, 2001–2006, 2007–2009, 2010–2014). X-axis: calendar periods, Y-axis: proportion of RHI in percentages.

Between 1996 and 2014 the date of HIV seroconversion could be estimated for 2608/12616 patients. Overall, 981/2608 (37.6%) patients were defined as RHI, with a trend for an increased proportion in latest years: from 213/578 (36.9%) in 1996–2000 up to 526/927 (56.7%) in more recent years (2010–2014; p<0.001) (Fig 1).

Table 1 shows the characteristics of the study population stratified by RHI status. RHI patients were younger than NRHI subjects (median age was 34, IQR 28–40 years in RHI and 36, IQR 31–42 in NRHI, p<0.001); proportion of females and risk factors for HIV transmission were similar between the two groups of patients. RHI patients also presented higher CD4+ T cells count in comparison with NRHI (CD4+ counts, cells/mmc: 493, IQR 336–667 in RHI and 452, IQR 289–632 in NRHI, p<0.001) (Table 1). Median time from the estimated date of seroconversion to entry in the cohort was 6 (IQR 4–9) in RHI and 27 (IQR 18–45) in NRHI (p<0.001).

**Table 1:** Characteristics of the study population according to RHI status.

	Recent seroconversion			
Characteristics	Yes (RHI)	No (NRHI)	p-value	Total
	N 981	N 1627		N 2608
***Age*, *years*, *median (IQR)***	34 (28, 40)	36 (31, 42)	<0.001	35 (30, 42)
***Females*, *n (%)***	117 (11.9%)	273 (16.8%)	1.000	390 (15.0%)
***Mode of HIV Transmission*, *n (%)***			0.501	
IDUs	79 (8.1%)	164 (10.1%)		243 (9.4%)
Homosexual contacts	587 (59.9%)	893 (55.3%)		1480 (57.0%)
Heterosexual contacts	256 (26.1%)	489 (30.1%)		745 (28.6%)
Other/Unknown	58 (5.9%)	70 (4.3%)		128 (4.9%)
***Not Italian nationality*, *n (%)***	123 (12.5%)	212 (13.0%)	0.050	335 (12.8%)
***AIDS diagnosis*, *n (%)***	26 (2.7%)	67 (4.1%)	0.478	93 (3.6%)
***CVD diagnosis*, *n (%)***	3 (0.3%)	8 (0.5%)	0.295	11 (0.4%)
***HBsAg*, *n (%)***			0.036	
Negative	947 (96.5%)	1582 (97.2%)		2529 (97.0%)
Positive	34 (3.5%)	45 (2.8%)		79 (3.0%)
Not tested	309 (31.5%)	553 (34.0%)		862 (33.1%)
***HCV Ab*, *n (%)***			0.161	
Negative	606 (61.8%)	932 (57.3%)		1538 (59.0%)
Positive	80 (8.2%)	172 (10.6%)		252 (9.7%)
Not tested	295 (30.1%)	523 (32.1%)		818 (31.4%)
***CD4 count*, *cells/mmc*,** ***median (IQR)***	493 (336, 667)	452 (289, 632)	<0.001	469 (307, 651)
***CD4 count nadir*, *cells/mmc*,** ***median (IQR)***	495 (338, 669)	452 (293, 625)	<0.001	470 (309, 641)
***CD8 count*, *cells/mmc*,** ***median (IQR)***	904 (654, 1284)	931 (688, 1283)	0.383	923 (675, 1283)
***Viral load*, *log***_***10***_ ***copies/mL*,** ***median (IQR)***	4.67 (4.06, 5.23)	4.42 (3.81, 4.97)	<0.001	4.5 (3.89–5.07)
***Time from HIV diagnosis to date of enrolment*, *months*,** ***median (IQR)***	6 (4, 9)	27 (18, 45)	<0.001	17 (8, 32)
***Site geographical position*, *n (%)***			0.017	
North	590 (60.1%)	916 (56.3%)		1506 (57.7%)
Center	313 (31.9%)	616 (37.9%)		929 (35.6%)
South	78 (8.0%)	95 (5.8%)		173 (6.6%)
***Diabetes*, *n (%)***	10 (1.0%)	16 (1.0%)	<0.001	26 (1.0%)
***Smoking*, *n (%)***			0.950	
No	327 (33.3%)	595 (36.6%)		922 (35.4%)
Yes	289 (29.5%)	592 (36.4%)		881 (33.8%)
Unknown	365 (37.2%)	440 (27.0%)		805 (30.9%)
***Total cholesterol*, *mg/dL*,** ***median (IQR)***	165 (143, 192)	164 (140, 188)	0.395	165 (141, 189)
***HDL cholesterol*, *mg/dL*,** ***median (IQR)***	42 (34, 48)	41 (33, 49)	0.673	41 (34, 48)
***EGFR (CKD_Epi formula)*, *ml/min/1*.*73m***^***2***^**, *median (IQR)***	4.67 (4.04, 5.17)	4.37 (3.63, 4.90)	<0.001	4.45 (3.77, 5)
***Blood glucose*, *mg/dL*,** ***median (IQR)***	86 (79, 92)	86 (80, 95)	0.037	86 (80, 94)
***Use of statins*, *n (%)***	5 (0.5%)	8 (0.5%)	0.353	13 (0.5%)
***Use of blood pressure lowering drugs*, *n (%)***	16 (1.6%)	35 (2.2%)	0.003	51 (2.0%)
***Education*, *n (%)***			<0.001	
Primary school	44 (4.5%)	55 (3.4%)		99 (3.8%)
Secondary school	194 (19.8%)	354 (21.8%)		548 (21.0%)
College	307 (31.3%)	580 (35.6%)		887 (34.0%)
University	157 (16.0%)	253 (15.6%)		410 (15.7%)
Other/Unknown	279 (28.4%)	385 (23.7%)		664 (25.5%)
***Employment*, *n (%)***			<0.001	
Unemployed	128 (13.0%)	194 (11.9%)		322 (12.3%)
Employed	454 (46.3%)	789 (48.5%)		1243 (47.7%)
Self-employed	138 (14.1%)	284 (17.5%)		422 (16.2%)
Occasional	31 (3.2%)	48 (3.0%)		79 (3.0%)
Student	63 (6.4%)	47 (2.9%)		110 (4.2%)
Retired	15 (1.5%)	35 (2.2%)		50 (1.9%)
Invalid	1 (0.1%)	2 (0.1%)		3 (0.1%)
Housewife	20 (2.0%)	46 (2.8%)		66 (2.5%)
Other/unknown	131 (13.4%)	182 (11.2%)		313 (12.0%)

LEGEND: Categorical data are presented as absolute numbers (percentages); p values for comparison of proportions between RHI and NRHI are by Chi-square test. Quantitative data are presented as median (Interquartile Range, IQR); p values for comparison of medians between RHI and NRHI are by Mann Whitney test. IDUs, Intravenous Drug Users; CVD, cardiovascular diseases; EGFR, glomerular filtration rate.

As regards the characteristics of patients enrolled in the study stratified by calendar period, in 2010–2014 patients with a known date of seroconversion presented a median age of 33 years (IQR: 27–40), median CD4+ count was 479 cells/mmc (IQR: 331–629) and HIV-RNA 4.65 log_10_ copies/mL (IQR: 4.11–5.16). With more recent years, we observed a reduction of females, AIDS presenters, hepatitis B and C co-infected patients and intravenous drug users (IDUs), but a sharp increase in men who have sex with men (MSM) and in people from northern Italy. Higher baseline HIV-RNA has also been reported in more recent calendar periods. Conversely, no difference in age and CD4+ count at enrolment was displayed (Table 2).

**Table 2:** Characteristics of patients with a known date of seroconversion according to calendar period.

	Period of Seroconversion					
Characteristics	1996–2000	2001–2006	2007–2009	2010–2014	p-value	Total
	N 578	N 676	N 427	N 927		N 2608
***Recent HIV infection*, *n (%)***	213 (36.9%)	122 (18%)	120 (28.1%)	526 (56.7%)	<0.001	981 (37.6%)
***Age*, *years–*** ***median (IQR)***	34 (30–41)	37 (31–43)	36 (29–42)	33 (27–40)	<0.001	35 (30, 42)
***Females*, *n (%)***	163 (28.2%)	108 (16%)	45 (10.5%)	74 (8%)	<0.001	390 (15.0%)
***Mode of HIV Transmission*, *n (%)***					<0.001	
IDUs	128 (22.1%)	62 (9.2%)	23 (5.5%)	30 (3.3%)		243 (9.4%)
Homosexual contacts	185 (32.0%)	376 (55.7%)	264 (62.6%)	655 (71.1%)		1480 (57.0%)
Heterosexual contacts	239 (41.3%)	207 (30.6%)	119 (27.9%)	180 (19.4%)		745 (28.6%)
Other/Unknown	26 (4.5%)	30 (4.4%)	16 (3.8%)	56 (6.1%)		128 (4.9%)
***Not Italian Nationality*, *n (%)***	40 (6.9%)	77 (11.4%)	65 (15.2%)	153 (16.5%)	0.449	335 (12.8%)
***AIDS diagnosis*, *n (%)***	33 (5.7%)	28 (4.1%)	19 (4.4%)	13 (1.4%)	<0.001	93 (3.6%)
***CVD diagnosis*, *n (%)***	2 (0.3%)	2 (0.3%)	5 (1.2%)	2 (0.2%)	0.073	11 (0.4%)
***HBsAg*, *n (%)***					<0.001	
Negative	547 (94.6%)	659 (97.5%)	419 (98.1%)	904 (97.5%)		2529 (97%)
Positive	31 (5.4%)	17 (2.5%)	8 (1.9%)	23 (2.5%)		79 (3%)
Not tested	143 (24.7%)	244 (36.1%)	160 (37.5%)	315 (34%)		862 (33.1%)
***HCV Ab*, *n (%)***					<0.001	
Negative	306 (52.9%)	367 (54.3%)	258 (60.4%)	607 (65.5%)		1538 (59%)
Positive	141 (24.4%)	60 (8.9%)	25 (5.9%)	26 (2.8%)		252 (9.7%)
Not tested	131 (22.7%)	249 (36.8%)	144 (33.7%)	294 (31.7%)		818 (31.4%)
***CD4 count*, *cells/mmc*,** ***median (IQR)***	480 (294, 670)	466 (301, 655)	448 (295, 643)	479 (331, 629)	0.566	469 (307, 651)
***CD4 count nadir*, *cells/mmc*,** ***median (IQR)***	473 (294, 664)	460 (301, 630)	448 (293, 617)	481 (337, 643)	0.079	470 (309, 641)
***CD8 count*, *cells/mmc*,** ***median (IQR)***	865 (657, 1196)	942 (678, 1277)	990 (728, 1428)	930 (658, 1340)	0.001	923 (675, 1283)
***Viral load*,** ***log***_***10***_ ***copies/mL*,** ***median (IQR)***	4.35 (3.73, 5.01)	4.42 (3.78, 5.00)	4.57 (3.98, 5.00)	4.65 (4.11, 5.16)	<0.001	4.50 (3.89, 5.07)
***Time from HIV diagnosis to date of enrolment*, *months*, *median (IQR)***	16 (8, 31)	33 (17, 62)	21 (11, 35)	10 (5, 18)	<0.001	17 (8, 32)
***Site geographical position*, *n (%)***					<0.001	
North	284 (49.1%)	357 (52.8%)	256 (60.0%)	609 (66%)		1506 (57%)
Center	225 (38.9%)	278 (41.1%)	153 (35.8%)	273 (29%)		929 (37%)
South	69 (11.9%)	41 (6.1%)	18 (4.2%)	45 (4.9%)		173 (6%)
***Diabetes*, *n (%)***	5 (0.9%)	6 (0.9%)	9 (2.1%)	6 (0.6%)	0.083	26 (1.0%)
***Smoking*, *n (%)***					<0.001	
No	100 (17.3%)	262 (38.8%)	169 (39.6%)	391 (42.2%)		922 (35.4%)
Yes	157 (27.2%)	265 (39.2%)	156 (36.5%)	303 (32.7%)		881 (33.8%)
Unknown	321 (55.5%)	149 (22.0%)	102 (23.9%)	233 (25.1%)		805 (30.9%)
***Total cholesterol*, *mg/dL*, *median (IQR)***	164 (141, 195)	162 (137, 187)	166 (141, 190)	165 (143, 189)	0.380	165 (141, 189)
***HDL cholesterol*, *mg/dL*, *median (IQR)***	40 (32, 48)	41 (33, 50)	42 (34, 48)	41 (34, 48)	0.785	41 (34, 48)
***EGFR*** ***(CKD_Epi formula)*, *ml/min/1*.*73m***^***2***^**, *median (IQR)***	4.20 (3.56, 5)	4.37 (3.63, 4.99)	4.52 (3.90, 4.91)	4.71 (4.04, 5.17)	0.002	4.45 (3.77, 5.00)
***Blood glucose*, *mg/dL*, *median (IQR)***	87 (81, 95)	86 (80, 93)	87 (80, 94)	85 (79, 93)	0.011	86 (80, 94)
***Use of statins*, *n (%)***	1 (0.2%)	5 (0.7%)	2 (0.5%)	5 (0.5%)	0.559	13 (0.5%)
***Use of blood pressure lowering drugs*, *n (%)***	3 (0.5%)	22 (3.3%)	9 (2.1%)	17 (1.8%)	0.006	51 (2.0%)
***Started ART over 3 months after enrolment*, *n (%)***	272 (47.1%)	255 (37.7%)	182 (42.6%)	458 (49.4%)	<0.001	1167 (44.7%)
***Education*, *n (%)***					<0.001	
Primary school	49 (8.5%)	18 (2.7%)	14 (3.3%)	18 (1.9%)		99 (3.8%)
Secondary school	201 (34.8%)	160 (23.7%)	67 (15.7%)	120 (12.9%)		548 (21.0%)
College	157 (27.2%)	247 (36.5%)	164 (38.4%)	319 (34.4%)		887 (34.0%)
University	43 (7.4%)	108 (16.0%)	68 (15.9%)	191 (20.6%)		410 (15.7%)
Other/Unknown	128 (22.1%)	143 (21.2%)	114 (26.7%)	279 (30.1%)		664 (25.5%)
***Employment*, *n (%)***					<0.001	
Unemployed	104 (18.0%)	80 (11.8%)	41 (9.6%)	97 (10.5%)		322 (12.3%)
Employed	260 (45.0%)	360 (53.3%)	204 (47.8%)	419 (45.2%)		1243 (47.7%)
Self-employed	107 (18.5%)	101 (14.9%)	77 (18.0%)	137 (14.8%)		422 (16.2%)
Occasional	25 (4.3%)	21 (3.1%)	11 (2.6%)	22 (2.4%)		79 (3.0%)
Student	13 (2.2%)	23 (3.4%)	13 (3.0%)	61 (6.6%)		110 (4.2%)
Retired	13 (2.2%)	21 (3.1%)	8 (1.9%)	8 (0.9%)		50 (1.9%)
Invalid	2 (0.3%)	0 (0.0%)	0 (0.0%)	1 (0.1%)		3 (0.1%)
Housewife	43 (7.4%)	13 (1.9%)	6 (1.4%)	4 (0.4%)		66 (2.5%)
Other/unknown	11 (1.9%)	57 (8.4%)	67 (15.7%)	178 (19.2%)		313 (12.0%)

LEGEND: Categorical data are presented as absolute numbers (percentages); p values for comparison of proportions among different calendar periods are by Chi-square test. Quantitative data are presented as median (Interquartile Range, IQR); p values for comparison of medians among different calendar period are by Kruskal-Wallis test. IDUs, Intravenous Drug Users–ART, HIV combination antiretroviral therapy, EGFR, glomerular filtration rate.

### Factors associated with Recent HIV Infections in Italy over 1996–2014

Factors associated with RHI by fitting a multivariable logistic regression analysis were younger age at HIV diagnosis, higher baseline CD4+ T-cells and HIV-RNA. More recent calendar period (2010–2014) was also associated with a 12-fold higher probability of RHI *versus* 1996–2000 (Table 3).

**Table 3:** Parameters associated with Recent HIV Infections (RHI) by univariate and multivariate logistic regression analysis.

	Odds ratios of recent seroconversion			
Characteristic	Unadjusted OR (95% CI)	p-value	Adjusted OR (95% CI)	p-value
***Calendar period of Seroconversion***				
1996–2000	1		1	
2001–2006	0.38 (0.29, 0.49)	<0.001	1.04 (0.67, 1.61)	0.858
2007–2009	0.67 (0.51, 0.88)	0.004	1.48 (0.91, 2.41)	0.115
2010–2014	2.25 (1.82, 2.78)	<0.001	12.01 (6.69, 21.57)	<0.001
***Gender***				
Female vs. male	0.67 (0.53, 0.85)	<0.001	0.66 (0.37, 1.20)	0.175
***Mode of HIV Transmission***				
IDUs	1		1	
Homosexual contacts	1.36 (1.02, 1.82)	0.034	1.08 (0.50, 2.31)	0.843
Heterosexual contacts	1.09 (0.80, 1.48)	0.596	0.90 (0.42, 1.94)	0.796
Other/Unknown	1.72 (1.11, 2.67)	0.016	1.86 (0.65, 5.35)	0.250
***Nationality***				
Not Italian vs. Italian	0.96 (0.75, 1.21)	0.717		
***AIDS diagnosis***				
Yes vs. No	0.63 (0.40, 1.00)	0.052	1.56 (0.64, 3.78)	0.328
***HCV Ab***				
Negative	1		1	
Positive	0.72 (0.54, 0.95)	0.021	0.82 (0.42, 1.61)	0.560
Not tested	0.87 (0.73, 1.03)	0.113	0.65 (0.42, 1.00)	0.050
***Age*, *years***				
per 10 years older	0.77 (0.69, 0.86)	<0.001	0.78 (0.65, 0.95)	0.011
***CD4 count*, *cells/mmc***				
per 100 cells higher	1.05 (1.02, 1.08)	0.003	1.09 (1.03, 1.16)	0.004
***CD8 count*, *cells/mmc***				
per 100 cells higher	1.00 (1.00, 1.01)	0.208		
***Viral load*, *log***_***10***_ ***copies/mL***				
per log copies/mL higher	1.34 (1.22, 1.48)	<0.001	1.41 (1.16, 1.71)	<0.001
***Diabetes***				
Yes vs. No	1.04 (0.47, 2.29)	0.928		
***Smoking***				
No	1		1	
Yes	0.89 (0.73, 1.08)	0.233	1.25 (0.88, 1.78)	0.219
Unknown	1.51 (1.24, 1.83)	<0.001	1.43 (0.84, 2.42)	0.185
***Total cholesterol*, *mg/dL***				
per 10 mg/dL higher	1.15 (0.93, 1.44)	0.204		
***HDL cholesterol*, *mg/dL***				
per 100 mg/dL higher	0.72 (0.31, 1.68)	0.447		
***Use of statins***				
Yes vs. No	1.04 (0.34, 3.18)	0.949		
***Use of blood pressure lowering drugs***				
Yes vs. No	0.75 (0.42, 1.37)	0.354		
***Blood glucose*, *mg/dL***				
per 100 mg/dL higher	0.44 (0.22, 0.85)	0.015	0.69 (0.24, 1.99)	0.490
***Site geographical position***				
North	1		1	
Center	0.79 (0.66, 0.94)	0.007	1.02 (0.71, 1.45)	0.930
South	1.27 (0.93, 1.75)	0.133	1.24 (0.66, 2.30)	0.505
***Education***				
University	1			
Primary/Secondary school	0.94 (0.73, 1.21)	0.622		
College	0.85 (0.67, 1.09)	0.199		
Other/Unknown	1.17 (0.91, 1.50)	0.227		
***Employment***				
Unemployed	1		1	
Employed	0.87 (0.68, 1.12)	0.286	0.71 (0.42, 1.19)	0.197
Self-employed	0.74 (0.54, 1.00)	0.047	0.82 (0.44, 1.54)	0.543
Occasional	0.98 (0.59, 1.62)	0.934	0.90 (0.37, 2.19)	0.814
Student	2.03 (1.31, 3.15)	0.002	0.89 (0.35, 2.27)	0.815
Retired/Invalid/Housewife	0.66 (0.42, 1.03)	0.068	1.00 (0.41, 2.42)	0.995
Other/unknown	1.09 (0.79, 1.50)	0.590	0.60 (0.28, 1.30)	0.197

LEGEND: Univariate and multivariate logistic regression analysis; OR: odds ratio– 95%CI, 95% confidence interval. IDUs, Intravenous Drug Users–cART, HIV combination antiretroviral therapy. Parameters included in the multivariable logistic regression model were calendar period of seroconversion, gender, mode of HIV transmission, age, HCV coinfection, AIDS diagnosis, CD4+ T-cells count and HIV-RNA (measured at enrolment), employment status, smoking, blood glucose and site geographical position.

### Estimates of cART initiation and of viro-immunological success in Recent and Less Recent HIV Infections

Among all patients included in the analysis, 49% of participants started cART by 3 months from seroconversion with no differences over time: 47.1% in 1996–2000, 49.4% in 2010–2014 (Table 2). There was also no difference in the 2-year cumulative probability of cART initiation between RHI and NRHI, regardless of calendar period (74.2% in RHI, 74.1% in NRHI, p = 0.73). The reasons for non cART initiation in the study population were the following: an HIV diagnosis within 6 months of enrolment (58%), no indication for cART (25% which is likely to be CD4+ count and guidelines driven), first access to care (13%), patients’ choice (2%), physician’s choice (2%) and other/unknown (1%).

The proportion of patients achieving a HIV-RNA ≤500 copies/mL at 6 months from the date of starting cART was similar in RHI and NRHI and stratifying by time periods (2008–2009: RHI 100%, NRHI 96%; 2010–2014: RHI 97%, NRHI 97%) (Table 4).

**Table 4:** Proportion of patients achieving a HIV-RNA ≤500 copies/mL at 6 months from the date of starting cART.

	Calendar Period of enrolment			
	1996–2000	2001–2007	2008–2009	2010–2015
**RHI**	6/8 (75%)	27/30 (90%)	20/20 (100%)	176/182 (97%)
**NRHI**	-	38/42 (90%)	41/43 (96%)	349/360 (97%)
**Total**	6/8 (75%)	65/72 (90%)	61/63 (97%)	525/542 (97%)

LEGEND: RHI, Recent HIV Infections; NRHI, Non Recent HIV Infections. Data are presented as absolute numbers (percentages).

Rates and hazards of virological success from fitting a Cox regression analysis were also similar in RHI (9.4 events/person-years of follow-up, PYFU 95%CI 8.15–10.84) *versus* NRHI (9.87 events/PYFU, 95%CI 8.93–10.91; HR 0.91 of RHI *versus* NRHI, 95%CI 0.76–1.09).

**Fig 2:**
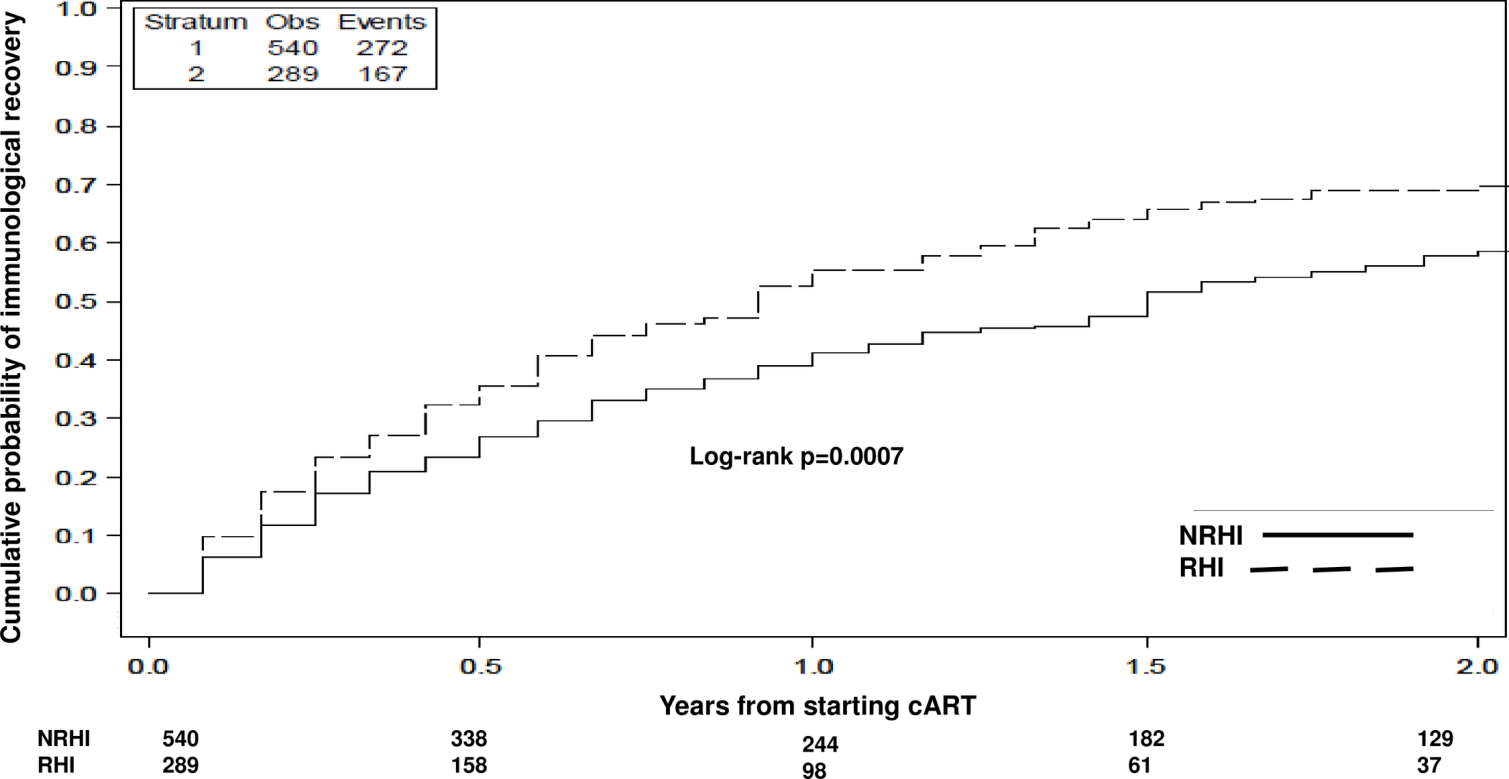
Probability of immune recovery (time from cART start to CD4+ count gain ≥200 cells/mmc) by Kaplan Meier estimates. Kaplan Meier estimates of the probability of achieving CD4+ T-cells count ≥200 cells/mmc from cART start according to Recent and non Recent HIV Infection; log rank test. The continuous line represents Less Recent HIV Infections (NRHI), the dot line represents Recent HIV Infections (RHI).

The Kaplan Meier estimates of immune recovery showed a higher probability for RHI compared to NRHI (log-rank test, p = 0.0007) (Fig 2).

In the Cox regression analysis, in fact, RHI showed a 1.5-fold higher probability of CD4+ gain ≥200 cells/mmc (RHI: 6.85 events/PYFU, 95%CI 5.79–8.1; NRHI: 5.79 events/PYFU, 95%CI 5.09–6.6; HR 1.46 *versus* NRHI, 95%CI 1.18–1.81), also following adjustment for calendar period and type of antiretroviral regimen started (adjusted HR 1.33, 95%CI 1.05–1.69) and for age, HCV co-infection and smoking (adjusted HR 1.44, 95%CI 1.09–1.9); the difference in probability was however largely attenuated after further controlling for baseline HIV-RNA and CD4+ count (adjusted HR 1.23, 95%CI 0.98–1.54). The results were similar also exploring the probability of immunological recovery according the second definition (CD4+ gain of 200 cells/mmc or reaching a single CD4+ count >350 cells/mmc, which ever came first): RHI, in comparison with NRHI, presented a higher probability of CD4+ gain by fitting Kaplan Meier curves (log-rank test p = 0.001) and Cox regression analysis (HR 1.26 *versus* NRHI, 95%CI 1.08–1.47), but the difference between RHI and NRHI was not confirmed after adjustment for calendar period, type of antiretroviral regimen, age, HCV coinfection, baseline HIV-RNA and CD4+ T cells count (adjusted HR 0.98 *versus* NRHI, 95%CI 0.74–1.31).

## Discussion

Trough the analysis of the data of untreated HIV-positive patients enrolled in an Italian cohort with documented dates of HIV-negative and positive serological tests, we tried to bridge the gap of lack of monitoring regarding the recent HIV infections. The knowledge of proportion and epidemiological features of new HIV infections is in fact essential in order to address screening services and try to control HIV epidemics. HIV test is the first step of the prevention interventions; in spite of the increased numbers of persons at risk of having HIV infection that are tested, still an unacceptable high proportion is not screened annually [16]. Since the awareness of HIV infection allows linkage to care, access to cART and virological suppression, with a reduced probability of HIV transmission, prevention measures are crucial to the reduction of HIV-related morbidity and mortality [17–19].

Among people in the ICONA cohort for whom the date of seroconversion could be accurately estimated, we hereby describe a significant trend for an increased proportion of RHI over time (57% in 2010–2014). This could be related to the increase in prevalence of MSM in recent years as well as an increase in self-awareness of risky behavior in this specific patient group [20, 21]. In our study we found no difference in mode of HIV transmission between RHI and NRHI; conversely, from 1996–2000 to most recent periods there was a decline in the proportion of IDUs and a significant increase in MSM (from 32% to 71%) in agreement with Italian data of the “Istituto Superiore di Sanità” and data of European Centre for Disease Prevention and Control (ECDC) about new HIV infections; the last report in fact described that in Italy the majority of new HIV infections were sexually acquired with 44.9% in heterosexuals and 40.6% in homosexuals [22, 23]. MSM are still vulnerable to HIV infection, as documented by the persistence of high prevalence of infection in this group, despite an overall decline of new infections in the general population [21, 24]. Data from the US Center of Disease Control and Prevention (CDC) report that 54% of estimated HIV diagnoses in United States in 2014 are in MSM, even if they represent only the 2% of the population [25]. A previous Italian study reported an increased risk of HIV seroconversion in younger MSM throughout the study period (1984–2010) [26]; similar data were confirmed in other European studies with a double risk of HIV in MSM aging 20–29 years from 2003 to 2012 [27, 28].

Our results are in keeping with the results from other European studies: the prevalence of recent infections varied from 7 to 47% due to differences in the prevalence of HIV infection in European countries, the analyzed study period and the definition of recent infection. All the other European studies described a shift in HIV risk from IDUs to sexual transmission in most recent years [29–31].

Policies of HIV prevention have changed over recent years acquiring new effective tools and targeting groups at risk in areas most affected by HIV epidemics. Our finding of an increased proportion of RHI might also reflect an increase of rate of HIV testing as a result of the introduction and implementation of ‘test and treat’ policies [32]. Currently, however, prevention services need to be monitored to identify weak areas to be improved. In fact, according to the last ECDC reports about HIV infection in the European Union, prevention interventions are still not enough to reduce the number of new HIV infections; in comparison with the UNAIDS 90:90:90 target, 15–17% of people living with HIV in Europe are estimated to have not yet been diagnosed, and among people diagnosed with HIV, nearly half are diagnosed late. Furthermore, 17% of people diagnosed with HIV are still not on treatment and the proportion of patients receving cART that are virally suppressed is around 51–95% [23].

By 3 months from seroconversion cART was started in half of the patients without significant differences between RHI and NRHI, but this analysis was performed before the results of the START trial became public [6].

The benefits of early treatment are now well recognized [33]: the 5-year risk of clinical progression is 3.2% in people starting cART immediately *versus* 7% in those deferring [34]. Rapid cART initiation is known to confer a significantly enhanced 2 year probability of immunological recovery, independently from baseline CD4+ count, and minimized HIV-associated inflammation [9]. These benefits translate in a reduced clinical progression in previous cohort studies [10, 11]. Similarly, retrospective studies have shown that long pretreatment waiting time in HIV-positive patients is associated with a higher risk of reduced cART adherence and subsequent higher mortality [35]. As previously demonstrated by HPTN052 trial, besides reducing AIDS and non AIDS related diseases, early cART initiation is also associated with a reduction of 96% in HIV transmission [18, 36]. Similarly, during a median follow-up of 1.3 years no cases of HIV transmission have been reported among serodifferent couples when the HIV-positive partner was under virally suppressive cART [18]. Our study reports data from 1996 to 2014; in the previous periods Italian guidelines suggested starting cART with a CD4+ count below 200 cells/mmc and then 350 cells/mmc. Most recent guidelines recommend that cART is offered to all HIV-positive patients, irrespective of CD4+ counts, also in order to reduce risk of transmission. Thus, in agreement with the UNAIDS 90:90:90 target [37], a reduction in HIV transmission is foreseeable that reflects the increasing number of HIV-positive patients on successful treatment.

Despite these data on clinical and immunologic advantages of treatment in the recent or acute phase [10, 11, 38], in our cohort we found similar rates of immune-virological response in RHI and NRHI; reasons are unclear, but it is possible that in the era of modern cART with highly effective and tolerated regimens differences are attenuated. Researchers of the Swiss HIV cohort showed that a substantial fraction of HIV transmissions can be attributed to recently infected patients, for whom the preventive effect of treatment is weaker, due to under-diagnosis and lack of patient’s awareness of their seropositive status [3]. Thus, early testing and the use of antiretroviral drugs associated with a rapid viral decay in RHI is crucial to maximize the effect of cART use as prevention and to reduce the risk of HIV transmission.

The main limitation of our analysis is the lack of differentiation between acute HIV and RHI (symptoms of acute infection are not collected in the cohort) and the potential selection bias introduced by including only cohort participants for whom the date of seroconversion could be accurately estimated; however, the increase of prevalence of MSM at enrolment has been also shown in analyses including the whole cohort. Furthermore, not all HIV-infected patients in Italy are enrolled in the ICONA cohort due to the design based upon voluntary enrolment. Finally, our finding of regional differences in the different calendar periods might reflect several changes in the centers participating to ICONA occurred in most recent years. Despite such limitations our data are in line with epidemiological reports by the “Istituto Superiore di Sanità” [22].

## Conclusions

The increased proportion of RHI over time in our cohort suggests that in recent years in Italy people are diagnosed earlier with HIV and more quickly enter care after the diagnosis. National data in fact show that the incidence of new HIV infections remained stable over the study period; nevertheless, efforts to the development and implementation of effective prevention interventions should continue to guarantee broad early cART access, reduce new infections and get closer to the UNAIDS 90-90-90 target.
